# High-Yield Characterization of Single Molecule Interactions with DeepTip^TM^ Atomic Force Microscopy Probes

**DOI:** 10.3390/molecules28010226

**Published:** 2022-12-27

**Authors:** Daniel Corregidor, Raquel Tabraue, Luis Colchero, Rafael Daza, Manuel Elices, Gustavo V. Guinea, José Pérez-Rigueiro

**Affiliations:** 1 Centro de Tecnología Biomédica, Universidad Politécnica de Madrid, 28223 Madrid, Spain; 2 Departamento de Ciencia de Materiales, ETSI Caminos, Canales y Puertos, Universidad Politécnica de Madrid, 28040 Madrid, Spain; 3 Centro de Investigación Biomédica en Red de Bioingeniería, Biomateriales y Nanomedicina (CIBER-BBN), Instituto de Salud Carlos III, 28029 Madrid, Spain; 4 Biomaterials and Regenerative Medicine Group, Instituto de Investigación Sanitaria del Hospital Clínico San Carlos (IdISSC), Calle Prof. Martín Lagos s/n, 28040 Madrid, Spain

**Keywords:** affinity atomic force microscopy, AFM, streptavidin, biotin, functionalization

## Abstract

Single molecule interactions between biotin and streptavidin were characterized with functionalized DeepTip^TM^ probes and used as a model system to develop a comprehensive methodology for the high-yield identification and analysis of single molecular events. The procedure comprises the covalent binding of the target molecule to a surface and of the sensing molecule to the DeepTip^TM^ probe, so that the interaction between both chemical species can be characterized by obtaining force–displacement curves in an atomic force microscope. It is shown that molecular resolution is consistently attained with a percentage of successful events higher than 90% of the total number of recorded curves, and a very low level of unspecific interactions. The combination of both features is a clear indication of the robustness and versatility of the proposed methodology.

## 1. Introduction

In recent years, the number of studies that apply atomic force microscopy (AFM) [1] to the study of biological systems has increased considerably. The interest in ascertaining the behaviour and characteristics of biomolecular interactions under physiological conditions makes this technique a great option due to the resolution it offers and the variety of tests it is possible to develop [2,3,4]. Among these tests, those that imply measuring intermolecular forces with a resolution of piconewtons, and those that perform a morphological characterization with nanometer resolution are of singular relevance [5,6]. However, and albeit sometimes overlooked, the study of these biological interactions and structures requires the use of chemically-functionalized AFM tips. The functionalization of the tips must allow the reliable attachment of the sensor molecules required in affinity (or chemical) atomic force microscopy which include, among others, antibodies and DNA single strands [7,8,9].

Currently, the commercial offer of functionalized cantilevers is very restricted and limited to a relatively reduced number of commercially available AFM tips in terms of materials, elastic constants, and resonance frequencies. Consequently, many research groups prefer to functionalize the tips adapted to their intended experiments following some of the available procedures developed to this end [10,11]. Several techniques are commonly used for functionalizing AFM tips, that can be broadly divided into liquid [12,13,14] and vapour functionalization techniques [12,15,16,17]. All these techniques employ molecules, such as tiolated compounds [18,19,20] and organometallics [21]. In particular, a significant fraction of the functionalization techniques relies on the molecule aminopropyltrietoxysilane (APTES) for the functionalization of Si and Si_3_N_4_ AFM tips [17,22,23,24,25,26,27].

However, these functionalization processes are extremely dependent on the chemistry of the surface [10] and, consequently, its implementation in the research laboratory tends to be hampered by the poor reproducibility of the outcome [28], which represents a major drawback for the performance of robust and reliable affinity atomic force spectroscopy experiments. This reduced reproducibility is a significant contribution to the low yield obtained in many studies, as reflected by percentages of successful events with respect to the total number of interactions undertaken typically lower than 10% [29,30]. As a consequence, it is customary to perform a number of experiments in the range of tens of thousands in order to obtain a few hundreds of validated F-d curves [31,32]

In this work, the interaction between biotin and streptavidin is used as model system, as commonly employed in affinity AFM studies [33,34,35,36,37], to show that DeepTip^TM^ probes, produced by the company Bioactive Surfaces S.L [38], can be easily decorated with sensor molecules following well-established cross-linking chemistries, and allow the consistent detection of single molecule events between biotin and streptavidin. It is shown that the DeepTip^TM^ probes may sustain repeated interactions between the sensor and the target streptavidin molecules, while not observing any sign of degradation in the tip. Lastly, a percentage of successful interactions of 90% with respect to the total number of interactions in the model system is obtained, which indicates the adequacy of these commercial AFM probes in combination with the developed methodology for high-yield applications in affinity microscopy experiments.

## 2. Results and Discussion

### 2.1. Amino Group Reactivity

**Figure 1:**
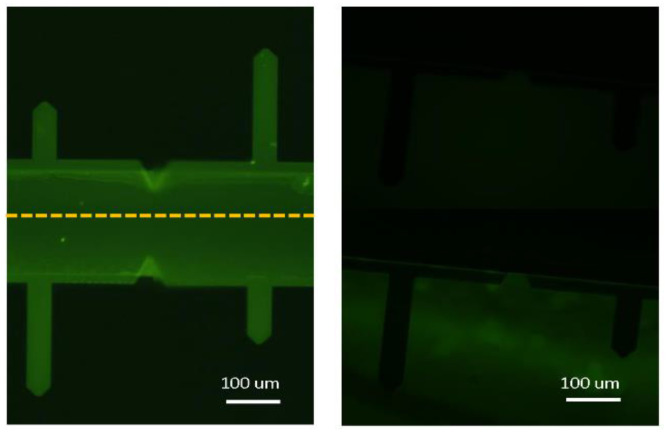
Fluorescent microscopy images of DeepTip^TM^ (**left**) and control probes (**right**). Samples were incubated with fluorescein isothiocyanate to assess the presence of amine groups at the surface. Images are composed to show both sides of the cantilevers.

The presence of reactive amine groups on the surface of the DeepTip^TM^ probes was assessed by fluorescence microscopy with the use of a fluorophore that reacts specifically with these groups. Figure 1 compares the fluorescence observed in functionalized probes with that of a control (non-functionalized samples). The increase in fluorescence of the functionalized samples indicates the presence of reactive amine groups on the surface of the functionalized probes.

### 2.2. Resonance Frequency and Elastic Constant

The resonance frequency and the elastic constant are two intrinsic parameters of AFM cantilevers, that depend both on the geometry and on the material of which the cantilevers are made, and determine the type of experiments for which the cantilevers are suitable.

The elastic constant and resonance frequency of 12 DeepTip^TM^ probes were characterized. Silicon nitride cantilevers with rectangular geometry and two different dimensions (100 × 40 μm and 200 × 40 μm) were analysed. Table 1 shows the results of resonance frequency and the elastic constant of both types of cantilevers, and represents a quantitative evidence of the high reproducibility in the properties of DeepTip^TM^ probes. Table 1 also compares the resonance frequency and elastic constant of the DeepTip probes with the original cantilevers prior to being functionalized. As expected, the functionalization process does not lead to any significant modification in the bulk properties of the probes.

### 2.3. Surface Topography and Roughness

**Figure 2:**
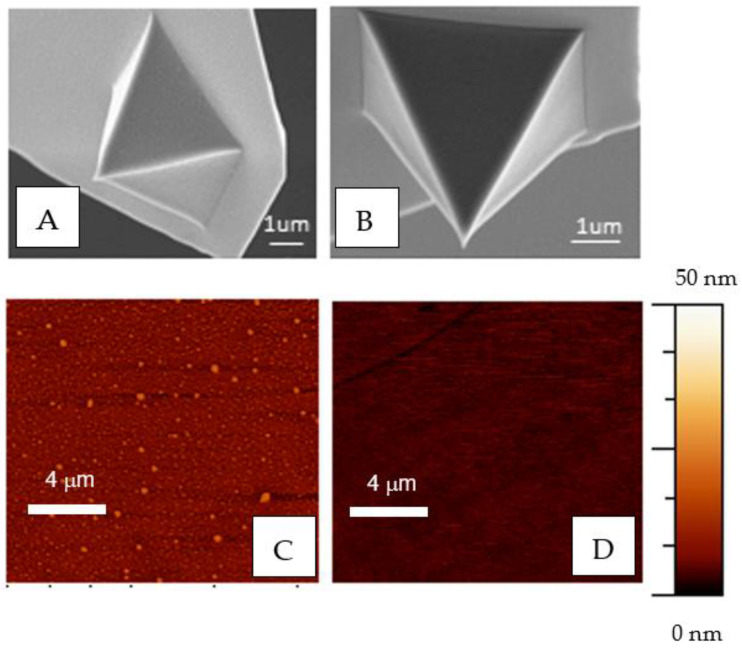
SEM images and AFM topography images of the DeepTip^TM^ probes. (**A**,**C**) functionalized tip, (**B**,**D**) non-functionalized tip (control).

The condition of the AFM tip is critical for the correct acquisition of the data and to avoid experimental artefacts. Therefore, the condition of the tips must be preserved if reproducible and reliable measurements are to be obtained. Figure 2A,C show, respectively, SEM micrographs of the tip and AFM micrographs of the surface of the cantilever from a functionalized cantilever and may be compared with those obtained from a control tip before being functionalized (Figure 2B,D). Although more quantitative details might be obtained by using a blind tip reconstruction technique [39], the images obtained with both microscopies do not indicate any significant modification of the cantilever when compared with the control, except for the appearance of small islands on the functionalized samples at nanoscale resolution. The topography of the probe was characterized quantitatively through the RMS parameter that yields a value of 2.8 nm for the DeepTip^TM^ probe in comparison with the 1.7 nm measured for the tip before being functionalized.

### 2.4. Biotin–Streptavidin Interaction Measured with the AFM

**Figure 3:**
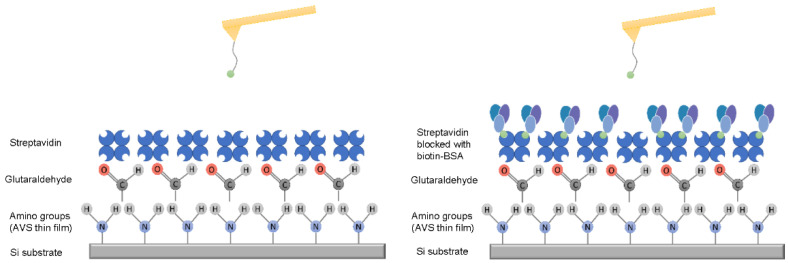
Schematic representation of the silicon substrate functionalized with streptavidin and of the AFM tip functionalized with the sensor molecule (biotin). (**Left**): non-blocked streptavidin. (**Right**): sample after the streptavidin molecules are blocked with biotin-BSA.

In order to assess the efficiency of the DeepTip^TM^ probes to perform experiments that imply molecular interactions, the biotin-streptavidin system was used as model. A schematic representation of the system is presented in Figure 3.

Streptavidin-coated silicon substrates were prepared as explained below, and experiments proceeded by recording 250 F-d curves with a single tip on a substrate. Supplementary Figure S1 Subsequently, the sample was incubated with biotin–albumin to block the binding sites of streptavidin, and 250 F-d additional curves were recorded in the blocked condition with the same tip. Consequently, each couple tip-substrate was used to obtain 500 curves. Since the obtaining of each curve takes approximately 15 s, the recording of the 250 curves of each batch is completed in a time of approximately one hour. The experiment was duplicated, so that the results presented below were obtained from two different DeepTip^TM^ probes on two different silicon substrates.

**Figure 4:**
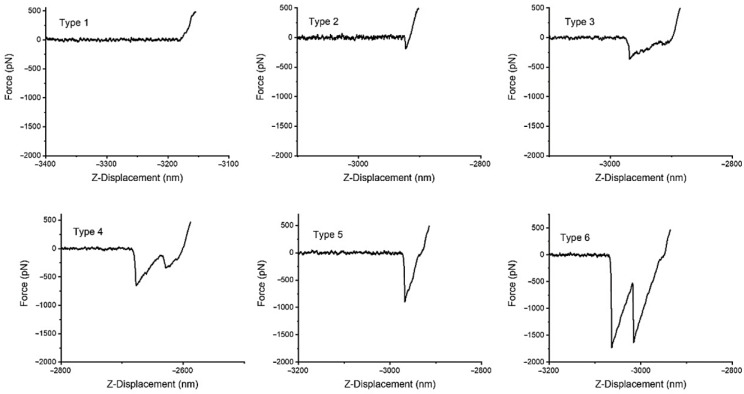
Representative curves of each of the six groups in which the interactions were classified. A seventh group was established with those curves considered as anomalous resulting from experimental artefacts.

All the curves were analysed to obtain both the adhesion force and the number of interactions (peaks). These parameters were used to classify the recorded curves into six different groups as illustrated in Figure 4.

The curves were classified by the following two criteria: (i) the maximum value of the adhesion force and (ii) the number of peaks present in the curve. Based on these two criteria, the following six groups were defined: (1) no interaction curves, in which no adhesion events are observed, (2) non-specific interaction curves, with low adhesion (upper limit of the adhesion force F = 200 pN), (3) single interaction curves, with a high adhesion event (upper limit of the adhesion force F = 650 pN), (4) multiple interactions–independent detachment curves, with several consecutive high adhesion events (upper limit of the adhesion force F = 650 pN), (5) multiple interactions–simultaneous detachment curves, with a very high adhesion event (upper limit of the adhesion force F = 2000 pN), and (6) multiple interactions–combined independent and simultaneous detachment curves, with a combination of multiple simultaneous and consecutive very high adhesion events (upper limit of the adhesion force F = 2000 pN). In addition, a seventh group that includes those curves that were discarded as experimental artefacts was also established. A representative curve of each one of the six groups is shown in Figure 4. It is worth indicating that establishing a quantitative criterium for the classification of the F-d curves allows the automatization of the procedure, so that a much larger number of curves can be conveniently classified in future works. However, the classification following this criterium of the curves indicated below proceeded manually, since the implementation of a fully automatized process will require a series of validation steps that were outside of the initial scope of this work.

**Figure 5:**
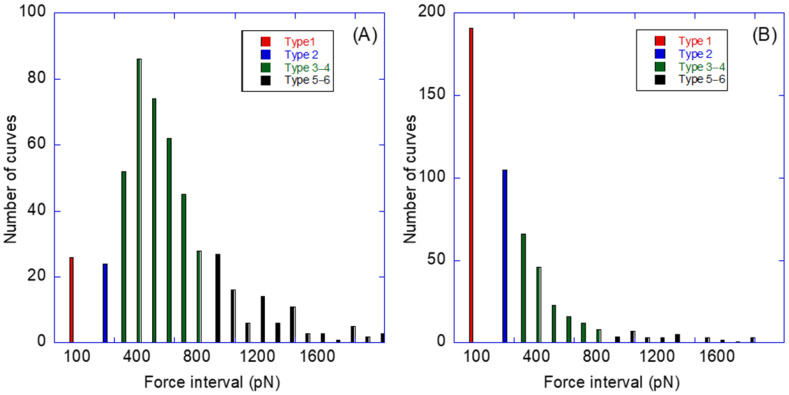
Histogram with the number of curves grouped by the value of the adhesion force of (**A**) pristine streptavidin and (**B**) blocked streptavidin. The range of forces that corresponds to each type of curve is indicated in the Figure.

**Figure 6:**
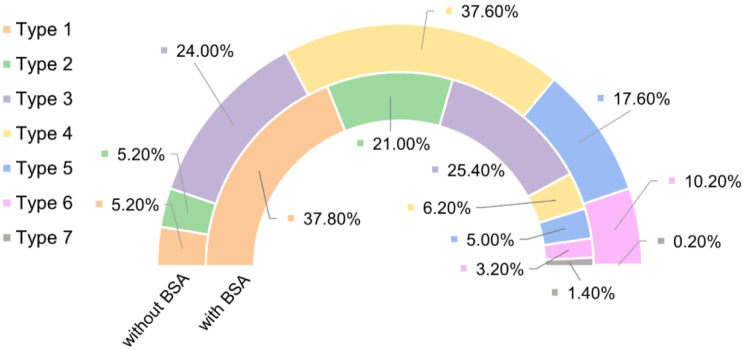
Chart with the percentages of each group of curves in both experimental conditions without (pristine streptavidin) and with BSA (blocked streptavidin). Type 1: no interaction; Type 2: non-specific interaction; Type 3: single interaction; Type 4: multiple interactions–independent detachment; Type 5: multiple interactions–simultaneous detachment; Type 6: multiple interactions–combined independent and simultaneous detachment; Type 7: discarded F-d curves.

Figure 5 shows the number of curves for each value of the adhesion force, and the distribution of relative frequencies of each type of curves under the two experimental conditions considered (pristine streptavidin and blocked streptavidin) is shown in Figure 6.

**Figure 7:**
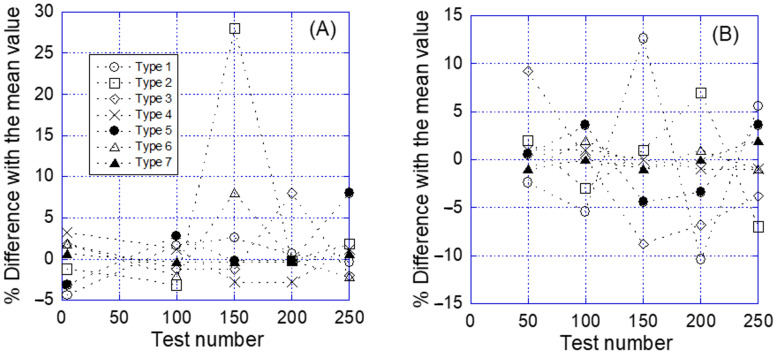
Difference of the percentage of curves that correspond to a given type as a function of the number of experiments for (**A**) experiments without BSA and (**B**) experiments with BSA. The % difference is calculated by subtracting the percentage of curves of a given type in an interval (i.e., curves from 51 to 100) from the arithmetic mean calculated from all the curves obtained in a given type of experiment.

In order to assess the robustness of the procedure and, especially, of the repetitive interaction between the functionalized tip and the substrate, the distribution of the curves in the seven types with respect to the order of each curve along the temporal series is shown in Figure 7. Figure 7 presents the difference in the percentage of a given type of curve in an interval of 50 consecutive curves along the temporal series and the arithmetic mean calculated for that type, considering the whole set of curves of a given experiment, either in the pristine condition or after incubation with biotin-BSA. In this case, the absence of any defined trend along the temporal series with respect to the number of curves belonging to a given group points to the robustness of the procedure, since it precludes any significant degradation of the tip as a result of the repetitive interaction with the sample.

The identification of a single molecular interaction between a sensor molecule and its target is the essential objective of affinity atomic force microscopy. In order to reach this objective, the experimental system must comply with three basic requirements: (a) no interaction should be measured in the absence of the target molecule, but (b) it must provide enough resolution to distinguish genuine molecular interactions between sensor and target molecules, and, additionally, (c) it must allow the repetition of the experiment in a series of at least hundreds (and probably thousands) of interaction events. As shown above, DeepTip^TM^ probes fulfil these three requirements when applied to the characterization of the interaction between biotin and streptavidin.

In this context, an event is considered to be successful if a sufficient adhesion force is recorded when there is sufficient certainty about the presence of the target molecule on the substrate. In the particular case of this work, a successful event is defined by the identification of an interaction event between, at least, a biotin and a streptavidin molecule. Consequently, the curves of the Types 3, 4, 5, and 6 are considered as successful events. On the contrary, an unsuccessful event corresponds to the recording of no or very low adhesion force when the presence on the ligand is expected. Thus, curves of Type 7 are always considered as unsuccessful events. With respect to the definition of successful events, a difference may be established between the experiments on the pristine streptavidin-coated surface and those performed after incubation with BSA. In the first set of experiments the surface is assumed to be coated with streptavidin, so that any event of Type 1 may be considered as an unsuccessful event. In contrast, blocking the streptavidin surface with BSA implies a significant reduction in the number of accessible streptavidin (ideally no accessible streptavidin molecules should be available after blocking with BSA, but this process does not have a 100% yield), so that Type 1 curves in this set of experiments do not necessarily correspond to an unsuccessful event.

Although it might be argued that some information may be retrieved from curves of Type 2, their analysis would require a much more detailed study that is beyond the scope of this work, so that these curves were not counted among the successful events.

With regard to the first requirement established above, the absence of any apparent interaction in a percentage of curves that reach a 37% after blocking the streptavidin on the substrate with biotin–albumin is a clear indication of the absence of a strong unspecific interaction between the functionalized tip and the substrate. Such an unspecific interaction would represent a serious experimental drawback for the analysis of streptavidin-biotin interactions, especially when single interactions are considered, since it would limit the resolution of the measurement. In all other curves (except for those excluded as experimental artefacts that amount to a proportion of approximately 1%) the interaction between the probe and the substrate may be identified and quantified by the adhesion force measured during the backward step of the F-d curve. It is also shown that the curves that reflect an interaction between the AFM tip and the substrate may be classified in five groups based on two experimental magnitudes—the *maximum adhesion force*, and the *number of adhesion events* (peaks) recorded during the backward movement.

The interpretation of each of these types may rely on both theoretical considerations and on the statistics of the experiments performed. The adhesion force in the streptavidin-biotin system has been characterized in previous works, and is assumed to range from values of F ≈ 200 pN to values of F ≈ 1000 pN [40], with an average value of approx. 400 pN for the interaction of one biotin with one of the streptavidin subunits, and was obtained from the comparison of experimental and molecular dynamics simulations [30]. Values in the range of 100 pN may result from defined pulling geometries of a given biotin–streptavidin interaction [30], but might also result from a reduced mechanical stability of the binding of the biotin to the tip [41]. In this regard, values in the range of 200 pN and below are consistently found when the functionalization with biotin of the AFM proceeds through non-covalent interactions [29,42], for example, when the cantilevers are coated with biotinylated bovine serum albumin (biotin-BSA) [31]. In addition, the influence of the loading rate on the quantitative values of the adhesion forces obtained in one experiment cannot be overlooked [30,32,43]. In contrast, a number of studies report adhesion forces in the range of 600 pN-1000 pN [33,40] and even as high as F = 2000 pN [44]. These values are consistent with the simulation of the maximum force required to desorb a streptavidin molecule from a solid substrate in an aqueous environment, estimated in F_ads_ ≈ 500 pN [45], which implies that the adhesion force between biotin and streptavidin should be, at least, of the order of this value.

With regard to the number of *interaction events*, some of the works that report the highest values of the adhesion force also present F-d curves in which the presence of several biotin–streptavidin interaction events that detach independently from one another may be recognized [33,40]. This kind of multiple events is to be expected for sufficiently high densities of biotin molecules on the tip and of streptavidin protein on the substrate. Besides, the independence of the detachment events also indicates that the different biotin (or equivalently, streptavidin) molecules involved in these interactions must be located at sufficient distance to justify this independence. It might be argued that the highest value of F = 2000 pN [44] may be the consequence of two biotin–streptavidin interactions sufficiently close to one another, so as to allow the simultaneous detachment of both pairs of interacting molecules. It is remarkable that the streptavidin–biotin interaction can be characterized with such precision with the experimental setup used in this work, since those data would be probably refined with the use of an elastic linker between the biotin and the AFM tip [46,47].

The results presented above, with respect to the classification of the interaction events in six groups and the proportion of each group found for each experiment, are consistent with the previous discussion. Thus, it may be argued that the single high adhesion events (Type 3) correspond to the single molecular interaction of one biotin molecule with one streptavidin molecule. Correspondingly, those curves classified as multiple high adhesion events–independent detachment (Type 4) would correspond to the interaction between two or more biotin molecules with two or more streptavidin molecules, separated by a sufficient distance, so that the detachment events may be observed individually. Those curves characterized by very high adhesion forces, i.e., adhesion forces in excess of 1000 pN (Type 5) would correspond to the interaction between more than one biotin-streptavidin pair, but with molecules located so close, that the detachment occurs simultaneously. Lastly, Type 6 curves would imply a combination of independent (Type 4) and simultaneous (Type 5) detachments of several biotin–streptavidin pairs in a single F-d curve.

This interpretation of the F-d curves is also consistent with the frequency with which each type appears depending on the substrate used; either pristine streptavidin-coated substrate, or streptavidin-blocked with biotin–albumin. In the initial experiment on the pristine streptavidin the most frequent type of curves corresponds to the multiple interaction with independent detachment (Type 4), consistently with the high density of immobilized molecules expected on both the AFM tip and the substrate. This type is followed in terms of frequency by the single interaction (Type 3), and then by multiple interactions, either with simultaneous detachment (Type 5) or with a combination of simultaneous and independent detachments (Type 6). It is also worth mentioning that the number of curves that do not show any interaction event (Type 1) and those that show an unspecific event (applying the definition used in this work) with an adhesion force below 200 pN just represent 10% of the total number of curves. Consequently, the usage of DeepTip^TM^ probes leads to a success rate of 90% when considered with respect to the total number of F-d curves recorded.

These proportions are consistently modified after blocking the streptavidin molecules with biotinylated albumin. In these experiments the major fraction of curves is consistent with Type 1 (no interaction) and the combination of Type 1 and Type 2 (unspecific interaction) amounts to more than 50% of the total number of curves. The next largest fraction of curves corresponds to the single molecular interaction (Type 3), as expected from the existence of some streptavidin molecules that are not adequately blocked after treating the substrate with biotinylated albumin. In this case, the inversion in the proportion of single events (Type 3) and multiple events with independent detachment (Type 4) may be explained by the presence of streptavidin molecules that remain isolated after blocking the substrate with albumin. A similar reduction in the proportion of curves that correspond to multiple events (Types 5 and 6) is observed in the substrates after blocking the streptavidin molecules. Additionally, it should be stressed that no systematic evolution on the number of curves of each type was found with the increasing number of tests, as shown in Figure 7, which hints to the robustness of the functionalization process employed to decorate the AFM tips with biotin molecules.

## 3. Materials and Methods

DeepTip^TM^ probes (silicon nitride, nominal resonance frequency 67 kHz–17 kHz; k = 0.48–0.06 N/m) were produced and supplied by the company Bioactive Surfaces S.L. [38]. 3-Aminopropyltriethoxysilane, (purity 99%), and AcroSeal^®^ was obtained from Acros Organics (Thermo Scientific Chemicals, Beerse, Geel, Belgium). Fluorescein 5(6)-isothiocyanate, 30% hydrogen peroxide solution, 50% glutaraldehyde solution in water, Streptavidin from *Streptomyces avidinii*, albumin biotinamidocaproyl-labeled bovine lyophilized powder, and poly(ethylene glycol)-(N-hydroxysuccinimide 5-pentanoate)-ether 2-(biotinylamino)ethane (MW = 10,000 Da, which corresponds to an approximate length for the spacer of 50 nm) were provided by Sigma-Aldrich (María de Molina, Madrid, Spain). An amount of 32% *w*/*w* ammonia solution was obtained from Scharlau (Sentmenat, Barcelona, Spain). Sodium dodecyl sulfate (SDS) was provided by Fisher Scientific (María de Molina, Madrid, Spain).

### 3.1. Sample Cleaning

Silicon samples (one side polished) were cut in squares of 5 × 5 mm samples and cleaned by immersion for 10 min in an HF:isopropanol (1:9) solution. Subsequently, silicon samples were sonicated at 50 °C in isopropanol for 15 min and, again, at 50 °C in acetone for 15 min. After being cleaned, silicon samples were dried with argon and stored in a p24 multiwell until being functionalized. Silicon samples were functionalized as described in [28] resulting in a high density of amine groups at the surface.

### 3.2. Characterization of Amino Groups on the Surface of the Cantilever

Functionalized cantilevers were incubated in 1 mL of a 0.25 mg/mL FITC solution in PBS (8 mM Na_2_HPO_4_, and 2 mM KH_2_PO_4_, 137 mM NaCl and 2.7 mM KCl pH 7.4) for 20 min. The samples were then immersed in a 10% SDS solution in PBS for 5 min, followed by immersion in PBS three times, 5 min each. Finally, the cantilevers were rinsed with deionized water and mounted on a glass slide for observation under the microscope.

A fluorescence microscope Leica DFC340FX was used to verify the presence of amine groups on the surface of the functionalized cantilevers. The parameters used for the observation were as follows: exposition time 1–5 s, gain 2.1, gamma 0.83, and magnification ×20. Nonfunctionalized cantilevers were used as a control. Fluorescence intensity was quantified using the ImageJ software (Windows 64-bit Java 8, NIH, USA).

### 3.3. Topography of the Surface

The functionalized cantilevers were characterised by scanning electron microscopy (SEM) and atomic force microscopy (AFM). A FESEM Auriga Zeiss operated at V = 5.0 kV was used to acquire the images. Before observation in the SEM, the samples were metalized with an EMITEC SC7620 (Quorum Tech, Laughton, East Sussex, U.K.) sputter coater (metallization parameters: 60 s and 18 mA).

The topography of the functionalized cantilevers, 3 samples for each functionalization condition, was characterized by a CERVANTES AFM (NANOTEC S.L., Tres Cantos, Madrid, Spain). The following scanning parameters were used: scan velocity 1 line/s, resolution 512 points, and image size 15 μm. The average height and the RMS value were calculated using the WSxN software [48].

### 3.4. Characterization of the Resonance Frequency and Elastic Constant

The resonance frequency and elastic constant of the DeepTip^TM^ probes were also measured with a CERVANTES AFM. Both the resonance frequency and the elastic constant were measured in air, and the elastic constant was estimated by applying the Sader’s method [49], which requires determining the resonance curve and the geometry and dimensions of the cantilever.

### 3.5. Affinity Microscopy Measurements with a Biotin-Streptavidin Model System

The interaction between biotin-decorated DeepTip^TM^ probes and streptavidin-coated silicon substrates was characterized through Force–distance (F-d) curves. Streptavidin-coated silicon substrates were prepared by covalently binding streptavidin to substrates functionalized as explained elsewhere [50]. Briefly, streptavidin was immobilized to the amine groups of the functionalized silicon by immersing the samples initially in 500 μL of a 25% glutaraldehyde (GA) solution for 30 min and, subsequently, adding 500 μL of a 0.5 mg/mL solution of streptavidin in PBS (8 mM Na_2_HPO_4_, 2 mM KH_2_PO_4_ 137 mM NaCl, 2.7 mM KCl pH 7.4) Samples were incubated with the streptavidin solution for 30 min. After completing the incubation step, the silicon substrates were rinsed several times with PBS to remove non-immobilized streptavidin.

Biotin–PEG–NHS molecules with an approximate molecular weight of Mw ≈ 10,000 Da were covalently immobilized to the amine groups of the DeepTip^TM^ probes. The cantilevers were initially immersed in 150 μL of PBS (8 mM Na_2_HPO_4_, 2 mM KH_2_PO_4_ 8 mM Na_2_HPO_4_, and 2 mM KH_2_PO_4_, 137 mM NaCl, 2.7 mM KCl, pH 8.5) as described in [51] for 1 h. Subsequently, AFM cantilevers were incubated in 100 μL of a 5.5 mg/mL biotin–PEG–NHS solution in DMSO for 30 min. Finally, the cantilevers were rinsed with ultrapure water and stored in PBS pH 7.4 at 4 °C until use (maximum 24 h).

Force–distance (F-d) curves were acquired in PBS (2 mL; 8 mM Na_2_HPO_4_, 2 mM KH_2_PO_4_, 137 mM NaCl, 2.7 mM KCl; pH 7.4) with a NANOLIFE AFM (NANOTEC S.L., Madrid, Spain). The conditions of the experiments were as follows: forward velocity 1000 nm/s, backward velocity 2600 nm/s, force relative limit 0.25 V (equivalent to approximately 500 pN), contact time 1 s, and time between curves 2 s. A total of 250 F-d curves were acquired initially on the streptavidin-coated Si substrates. Subsequently, the silicon substrates were incubated for 30 min with 1 mL of 0.5 mg/mL solution of biotin–albumin in PBS (8 mM Na_2_HPO_4_, 2 mM KH_2_PO_4_, 137 mM NaCl, 2.7 mM KCl; pH 7.4) to block the binding sites of the streptavidin molecules, and 250 additional F-d curves were obtained with the same AFM tip. The experiment was duplicated with a different Si substrate and functionalized AFM tip for each duplicate.

## 4. Conclusions

Functionalized DeepTip^TM^ probes produced by the company Bioactive Surfaces S.L. have shown their ability to covalently bind sensor molecules that can be employed in affinity atomic force microscopy for the recognition of their target molecules, as demonstrated by its application to the characterization of the biotin–streptavidin interaction system. Biotin-functionalized tips can sustain up to 500 repetitive interactions with a substrate without exhibiting any sign of degradation of the tip itself, or of the sensor molecules. In addition, it is shown that biotinylated tips show a negligible interaction with substrates that do not contain any exposed streptavidin molecules. In contrast, the biotin-streptavidin interaction is clearly measured from the F-d curves.

The analysis of the F-d curves led to the classification of the curves in six different groups (and a seventh group that includes the small number of curves that are considered as experimental artefacts): Type 1 (no interaction), Type 2 (unspecific interaction), Type 3 (single molecular interaction), Type 4 (multiple molecular interaction with independent detachment), Type 5 (multiple molecular interaction with simultaneous detachment), and Type 6 (multiple molecular interaction with both simultaneous and independent detachments). The analysis of the frequency of each type of curves indicates that 90% of the total number of interactions leads to F-d curves that can be used to characterize the properties of the system. This large proportion of successful interaction events indicates that DeepTip^TM^ probes are extremely suitable candidates for a wide range of techniques in the framework of affinity microscopy. Since the reproducibility and stability of AFM singlemolecule F-d studies has long been questioned, the availability of a commercial probe that can sustain this remarkable success rate may represent a significant thrust for the application of this promising technique to various fields in biology, biomedicine, and materials science.

## Figures and Tables

**Table 1:** Resonance frequency and elastic constant values of the DeepTip^TM^ probes with different dimensions. The values corresponding to the probes before being functionalized are also shown to allow comparison.

Dimensions	Resonance Frequency (Hz)	Elastic Constant (N/m)
100 × 40 μm (DeepTip)	45,200 ± 400	0.18 ± 0.01
200 × 40 μm (DeepTip)	12,550 ± 50	0.026 ± 0.001
100 × 40 μm (Control)	46,700 ± 100	0.22 ± 0.01
200 × 40 μm (Control)	12,740 ± 30	0.032 ± 0.002

## Data Availability

Data are available upon request to the corresponding author.

## Supplementary Materials

The following supporting information can be downloaded at: https://www.mdpi.com/article/10.3390/molecules28010226/s1, Figure S1: Example of a curve discarded from the analysis as anomalous.

## Sample Availability

Samples of the compounds may be available upon request to the corresponding author.
