# Supplementary material for: High-Yield Characterization of Single Molecule Interactions with DeepTipTM Atomic Force Microscopy Probes
Source: Molecules. 2022 Dec 27;28(1):226. doi: 10.3390/molecules28010226 (PMC9822271; doi:10.3390/molecules28010226)
Supplement: Supplementary file 1 [file molecules-28-00226-s001.zip › molecules-2105794-supplementary.pdf]

Supplementary Material

High-Yield Characterization of Single Molecule Interactions with DeepTip™ Atomic Force Microscopy Probes

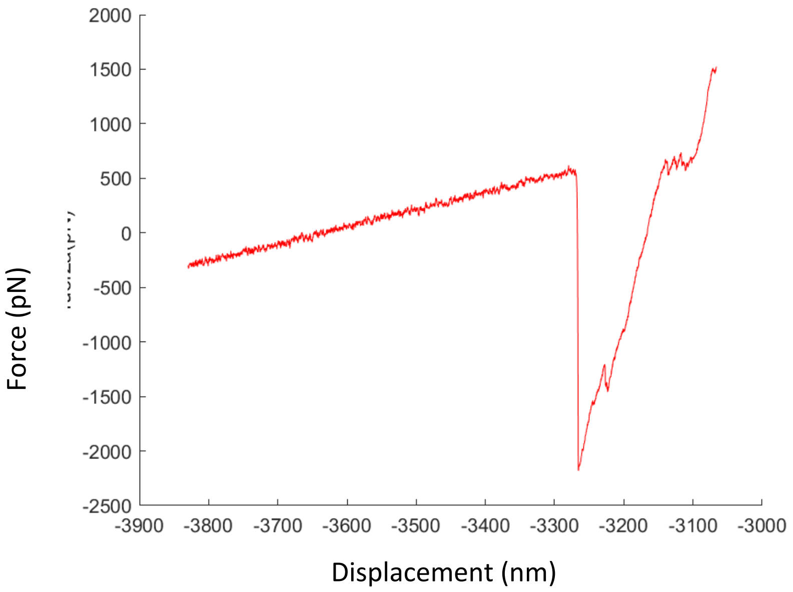

Supplementary Figure S1. Example of a curve discarded from the analysis as anomolous.
